# Pilot study demonstrating safety and efficacy of intravenous allogeneic uterine-derived mesenchymal stromal cell therapy in cats with naturally occurring osteoarthritis

**DOI:** 10.3389/fvets.2026.1900571

**Published:** 2026-09-04

**Authors:** Valentine Williams, Lilly Mick, Rebecca Windsor, Rudolph S. Parrish, Caryn M. Thompson, Emily Sundman, Carolyn Wrightson, Nopmanee Taechangam, Orzala Sharif, Linda Black

**Affiliations:** 1 Gallant, San Diego, CA, United States; 2 Vista Research, Bath, ME, United States

**Keywords:** feline, mesenchymal stem cell, osteoarthritis, pain, regenerative medicine

## Abstract

**Introduction:**

The objective of this study was to evaluate the tolerability and efficacy at 3 months of intravenous (IV) allogeneic uterine-derived mesenchymal stromal cells (UMSCs) for osteoarthritis (OA) in client-owned cats.

**Methods:**

This was a randomized, masked, placebo-controlled pilot study conducted across six veterinary clinics. Thirty-five client-owned cats with naturally occurring OA were randomly assigned to receive two intravenous injections, given 14 days apart, of one of three treatments: 5 million (5M) UMSCs, 20 million (20M) UMSCs, or saline placebo. Client-Specific Outcome Measures (CSOM), Owner Overall Assessment (OOA), Veterinarian Overall Assessment (VOA) and Veterinarian Pain Assessment (VPA) were recorded at day 0, 14, 30, 60 and 90.

**Results:**

At day 90, predefined treatment success was achieved in: CSOM and OOA for 7/9(78%) 5M UMSC and 10/12(83%) 20M UMSC treated cats compared to 4/11(36.4%) placebo-treated cats for both outcomes; VOA for 4/8(50%) 5M UMSC and 8/12(67%) 20M UMSC treated cats compared to 2/10(20%) placebo-treated cats; and VPA for 6/8(75%) 5M UMSC and 10/12(83%) 20M UMSC treated cats compared to 5/11(45%) placebo-treated cats. Treatment-related adverse events were observed in 1/10 (10%) 5M UMSC treated cats, 5/13 (38%) 20M UMSC treated cats, and 2/12 (16.7%) saline treated cats. The most common treatment related events reported in the first 24 hours were transient vomiting or nausea, weakness, lethargy, hypersalivation, tachypnea and malodorous breath.

**Discussion:**

This study demonstrated that IV administration of allogeneic UMSCs was well tolerated and showed improvement in owner and veterinarian outcomes at both 5M UMSC and 20M UMSC dosing to at least 3 months in cats with naturally occurring OA. Intravenous MSC therapy shows promise for cats with OA and warrants further exploration.

## 1 Introduction

Osteoarthritis (OA) is most frequently diagnosed in cats older than 12 years of age; however, the disease process typically starts much earlier in life, and radiographic evidence of OA has been demonstrated in greater than 60% of cats younger than 6 years of age (1–3). Despite this high prevalence, feline OA remains underdiagnosed, as clinical signs are often subtle and include behavioral changes, reduced activity, hesitancy to jump, and altered grooming habits (4). Observed lameness is rare (4).

Systemic biomarker studies describe an immune-mediated component in the pathophysiology of feline OA. Elevated systemic proinflammatory serum IL-2 and TNF- α cytokine levels have been reported in cats with greater radiographic OA severity and higher orthopedic pain scores (5). Transcriptomic profiling in cats with OA has revealed differential gene expression in pathways involved in major histocompatibility complex (MHC) molecule-mediated antigen presentation, T-cell receptor signaling, and oxidative phosphorylation (6). More recently, whole blood and serum proteomic analysis of cats with OA and OA-associated pain has demonstrated alterations in systemic proteins associated with acute phase response signaling and upregulation of complement system components among others (7). These findings underscore a need for therapeutics that target immune dysregulation as a key factor in the pathophysiology of OA in cats.

Current therapeutics focus on pain management and may be associated with off-target side effects leaving an unmet medical need in cats with OA (1, 8–11). Mesenchymal stromal cells (MSCs) have emerged as a potential therapy for a variety of feline diseases due to their anti-inflammatory and immunomodulatory properties (12–17). MSCs also offer the potential for disease modification by ameliorating the systemic and local immune dysregulation associated with OA and showing inherent chondrogenic ability, a key potential advantage in OA (18–23). Intravenously administered MSCs are reported to be well tolerated and have shown promising efficacy for several feline indications (15, 24), and specifically for OA in cats (25) and dogs (26–28).

The objective of this investigational randomized, masked, placebo-controlled pilot study was to evaluate tolerability and compare the efficacy of a two-dose regimen of 5 million (5 M) or 20 million (20 M) intravenously administered allogeneic uterine-derived MSCs (UMSCs) to saline (placebo) in client-owned cats with naturally occurring OA to inform dosing design for future studies. We hypothesized that UMSC therapy would be well tolerated and would result in improved owner and veterinarian-reported assessment scores compared with placebo and that efficacy outcomes in the 5 M and 20 M groups would be similar.

## 2 Materials and methods

### 2.1 Mesenchymal stromal cell preparation and characterization

Cells were isolated from uterine tissue obtained during routine ovariohysterectomy of a healthy, pathogen-free, FDA-qualified, feline donor. FDA qualified is an agreed upon donor program that classifies sourcing, rearing, maintaining, uterine-collection and processing, and relevant disease agent testing in accordance with CVM GFI #218: Cell-Based Products for Animal Use, CVM GFI #253: Current Good Manufacturing Practice for Animal Cells, Tissues, and Cell- and Tissue-Based Products and CVM GI #254: Donor Eligibility for Animal Cells, Tissues, and Cell- and Tissue-Based Products). Prior to harvesting, the donor was screened to exclude the presence of infectious disease agents and to confirm overall systemic health. The donor tissue was shipped by courier to the processing laboratory in a sterile receptacle and expanded under current good manufacturing practices (cGMP). In brief, the tissue was transferred into a biological safety cabinet, ovaries were resected and discarded, and the uterine tissue was manually dissected, minced, and digested with collagenase (VitaCyte®, Indianapolis, IN, USA). Cell culturing was performed in a proprietary optimized culture medium for feline UMSCs containing fetal bovine serum and carried out to passage six on flatware before cryopreservation.

**Figure 1:**
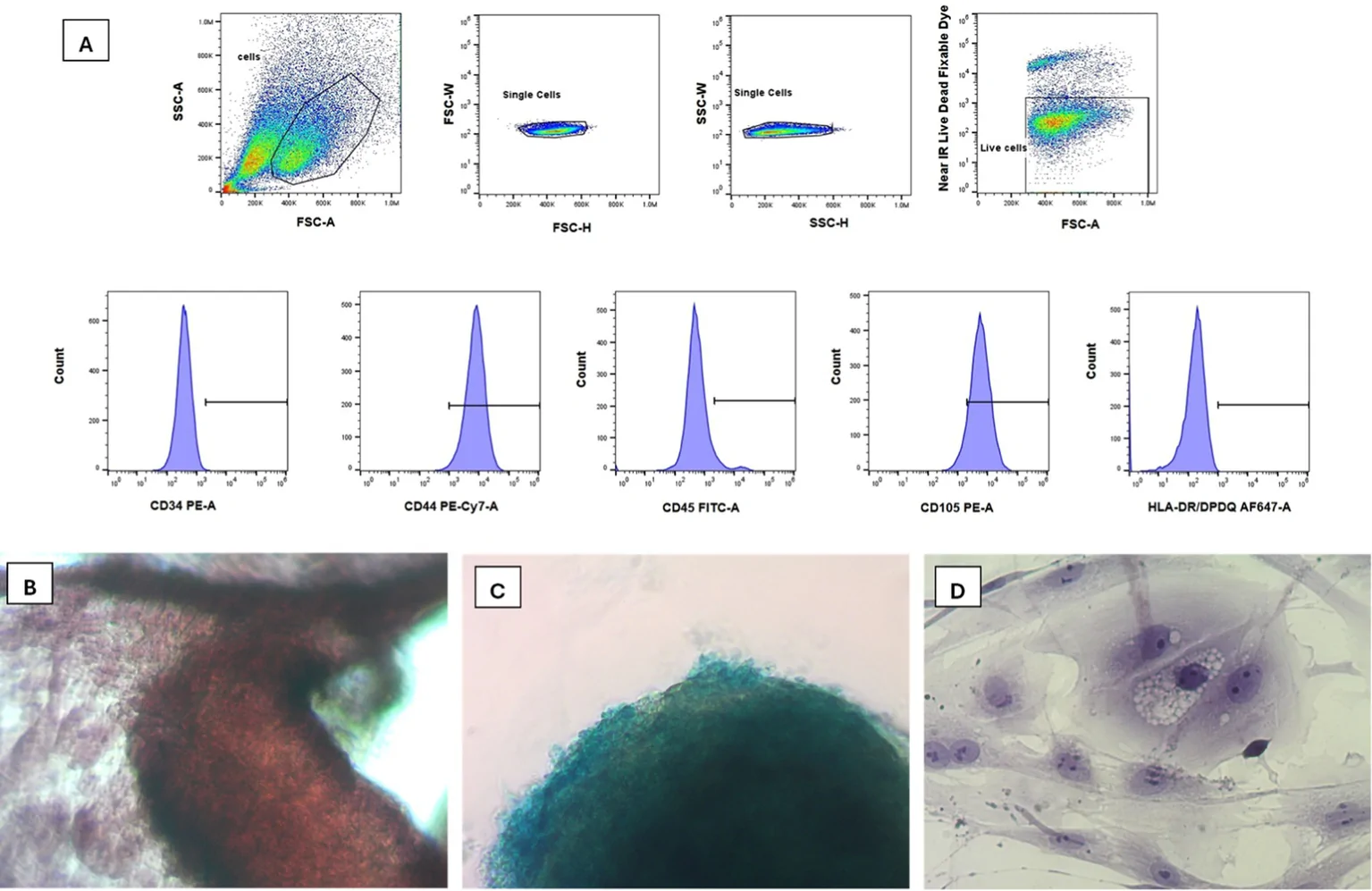
Immunophenotyping and trilineage differentiation of uterine-derived mesenchymal stromal cells (UMSCs). **(A)** Single-color immunophenotyping on UMSCs – gated on single live cells demonstrating CD34-, CD44+, CD45-, CD105+, and MHC II-(HLA-DR). **(B–D)** Trilineage differentiation staining on feline UMSCs working cell bank, showing **(B)** osteogenic differentiation through alizarin red staining, **(C)** chondrogenic differentiation through toluidine blue staining, and **(D)** adipogenic differentiation through oil red O staining with intracellular fat vacuoles. Image taken at 200x magnification on day 21 trilineage differentiation UMSCs.

Cells were analyzed with commercially available, directly labeled antibodies reactive with CD34, CD45, CD44, CD105, MHC-class II (ThermoFisher Scientific), and 7-aminoactinomycin D red fluorescent live/dead stain (7-AAD, Invitrogen, Carlsbad, CA, USA). The cells were incubated in the previously optimized reagents for 30 min in darkness and on ice, prior to being assessed for reactivity on Attune Flow Cytometer (Thermo Fisher), with data analyzed on FlowJo (Becton Dickinson, Ashland, OR, USA). The isolated feline UMSCs were assessed to be CD34-negative, CD44-positive, CD45-negative, CD105-positive and MHC-class II-negative prior to clinical use and multipotency was confirmed through trilineage differentiation assay prior to clinical administration (Figure 1). Cells tested negative for infectious agents.

Cells were transferred into 2 mL vials with septum containing 20 million viable UMSCs, frozen in a cryoprotectant excipient containing dimethyl sulfoxide (DMSO), and stored in vapor phase LN2 at −80 °C or below at the GMP manufacturing facility until the time of shipment. The cells were shipped in a validated shipper to the clinical sites overnight with enough dry ice to maintain a consistent temperature for up to 96 h and intended to be used upon receipt or stored in a − 80 °C Ultra Cold freezer. Data loggers were placed in all shippers, and there were no temperature excursions that would constitute potential compromise of the product.

### 2.2 Study design

This was a randomized, masked, placebo-controlled pilot study conducted at six veterinary clinics across the United States under Good Clinical Practice (GCP) guidelines (CVM GFI#85 – VICH GL9). The study aimed to enroll approximately 10 cats in each dose group (20 UMSC cats total) and 10 saline control cats to evaluate tolerability and compare efficacy signal between the three groups. The study protocol was reviewed and approved by an independent ethics review board (Veterinary Clinical Studies Committee, San Diego, CA) (29) prior to initiation. Written informed consent was obtained from all owners before any study-specific procedures were performed.

#### 2.2.1 Patient eligibility

Inclusion and exclusion criteria are listed below:

##### 2.2.1.1 Inclusion criteria

- Client-owned cats of any sex or reproductive status (intact or spayed/neutered), not owned by study personnel
- Was at least 6 months of age
- Was at least 1.0 kg body weight
- Was not breeding, pregnant or lactating
- Had radiographs in the last 90 days that confirmed OA in at least two joints or spinal segments
- Had evidence of pain or discomfort in at least one of the identified joints or spinal segments with radiographic evidence of OA as evidenced by pain on palpation or other means of clinical assessment
- Had no joint disease not associated with naturally occurring OA including but not limited to hip dysplasia, intervertebral disc disease, infectious joint disease, and/or immune-mediated joint disease
- Had no suspected or confirmed soft tissue disruption that affected the outcome assessment including ligament and tendon tears or ruptures and meniscal injuries
- Had no evidence of end-stage OA characterized by marked enthesiophytes, collapse of the joint space, or bridging ankylosis on imaging
- Was at least 6 months post-operative, if applicable, for joint disease with no evidence of joint instability
- Minimum total CSOM component score of 5 at Day 0 with only one CSOM score of 1 (mildly affected). No CSOM component score of 0 (normal) at screening or Day 0
- Had no known or suspected concurrent uncontrolled or medically unstable disease
- Non-steroidal anti-inflammatory was discontinued for at least 7 days prior to enrollment
- Monoclonal antibody analgesics discontinued for at least 30 days prior to enrollment
- Immunosuppressant drugs including corticosteroids were discontinued for at least 14 days prior to enrollment
- Was not treated with therapeutics that could have interfered with study objectives unless they had been consistently administered for at least 6 weeks prior to enrollment and no changes to the dose or frequency were allowed during the study
- Had a negative Feline Leukemia virus (FeLV) and Feline Immunodeficiency (FIV) test at screening
- No vaccinations were given within 14 days of screening or at the screening visit and no vaccinations were anticipated to be needed between the screening and Day 90 visits

##### 2.2.1.2 Exclusion criteria

- Was not expected to survive to the 90-day endpoint
- Was diagnosed with neoplasia that impacted musculoskeletal function or pain, was out of remission or would not remain in remission for duration of study
- Had been diagnosed with cranial cruciate ligament rupture ≤6 months prior to screening.
- Had joints with fractures or osteochondral fragments.
- Had joint lesion or severity that was an indication for surgery
- Had an unstable joint
- Had joint surgery in the last six (6) months prior to screening
- Had neurologic dysfunction or disease
- Was being treated with therapeutics that could have interfered with study objectives and assessments
- Had participated in another research study within 30 days prior to enrollment.
- Had a history of previously receiving stem cell therapy

#### 2.2.2 Concurrent medication use

Prior to screening, cats were withdrawn from medications that could potentially impact efficacy assessment. Withdrawal times were determined for each class of therapeutics based on known pharmacokinetic properties and included: at least 7 days for non-steroidal anti-inflammatory drugs (NSAIDs), 30 days for anti-NGF monoclonal antibody analgesics (frunevetmab), and 14 days for immunosuppressant drugs including corticosteroids (30–32). The following medications were prohibited for the duration of the study: analgesics including gabapentin, pregabalin, and opioids (unless required for sedation prior to IV catheter placement), anti-NGF monoclonal antibodies, NSAIDs, corticosteroids and appetite stimulants. There were no vaccinations allowed during the 90-day study.

#### 2.2.3 Screening assessments

For each cat, the same owner completed all owner assessments via an electronic mobile application (ePRO). Client-Specific Outcome Measures (CSOM), a validated, individualized, and subjective scoring system for OA (33, 34), was calculated by owners’ selection of three specific, unique activities for their cat that had become impaired since the onset of OA, evaluating each on a 5-point scale (0 = no problem, 1 = mildly problematic, 2 = moderately problematic, 3 = severely problematic, 4 = impossible) for a maximum possible total score of 12. At screening, cats had to have a minimum total CSOM score of 5. No individual CSOM activity metric could have a score of 0 (normal), and only one individual CSOM activity metric could have a score of 1 (mildly affected). Treatment success was defined as a reduction of at least two points in total CSOM score compared to baseline, with no increase in any individual activity (35).

All examinations for each cat were performed by the same masked veterinarian (clinical investigator) at each visit that had no association with the Sponsor. Veterinarian assessment of pain was conducted by palpating at least two pre-identified affected appendicular joints or spinal segments. Grading of the pain response was on a five point categorical scale: 1 (no resentment, normal amount of moving), 2 (mild withdrawal, mildly resists), 3 (moderate withdrawal, body tenses, may orient to site, may vocalize or increased in vocalization), 4 (orients to site, forcible withdrawal from manipulation, may vocalize, hiss or bite) and 5 (tried to escape, prevent manipulation, bite or hiss, marked guarding of area) (33, 36). Treatment success was defined as a one point or greater reduction in pain score in at least one joint or spinal segment without an increase in pain score in any other joint or spinal segment, a per-segment criterion selected to capture localized therapeutic effect that might not be reflected in a total summed score.

### 2.3 Treatment protocol

Eligible cats were randomly assigned to one of three groups in a 1:1:1 ratio using a block randomization algorithm within an electronic clinical trial management system (VISION; Prelude). Cats were assigned to receive a 5 M UMSCs, 20 M UMSCs, or saline (placebo) based on previously published dose ranges of IV MSC therapy for other feline indications (12, 15–17, 37). Veterinarians, owners, and all other study personnel evaluating the cats were masked to treatment assignment. A designated unmasked treatment administrator at each site was solely responsible for handling, thawing and preparing the treatments. Opaque masking labels were applied to the UMSC and placebo dosing syringes to maintain masking during the injection.

To facilitate IV catheter placement, mild sedation was permitted as needed at the discretion of the veterinarian with guidelines including trazodone 7–10 mg/kg PO 2 h prior to visit, gabapentin 7–10 mg/kg PO or transdermal 1–2 h prior to the visit, or butorphanol 0.3–0.4 mg/kg IM 20 min prior to catheter placement. Acepromazine and alpha-2 agonists were not recommended. Cats recovered from sedation prior to treatment administration to allow for appropriate safety observations. At the veterinarian’s discretion, premedication with diphenhydramine, maropitant, or both was allowed prior to treatment administration.

On days 0 and 14, cats received their randomized treatment via a 22-gage or larger intravenous catheter placed in the cephalic vein. Each container of cells was thawed at room temperature over 45 min. For the 5 M and 20 M UMSC dose groups, 0.5 mL and 2.0 mL of UMSCs, respectively, were drawn into a syringe and diluted with sterile 0.9% sodium chloride (saline) to a total volume of 10.0 mL. The placebo group was the same volume (10.0 mL) of sterile saline alone. All injections were administered slowly over 2 to 5 min, followed by a 0.5- to 1.0-mL saline flush.

### 2.4 Safety assessment

Cats were monitored continuously for 2 h following treatment and any clinical abnormalities were recorded if observed. Vitals (heart rate, respiratory rate, and rectal temperature) were assessed and recorded every 15 min. Injection sites were evaluated during treatment administration and at all *post*-treatment visits.

Clinical abnormalities observed in the post-treatment observation period or at any other point throughout the study were reported as adverse events (AEs). An AE was defined as any unfavorable and unintended observation or undesirable experience occurring after treatment, regardless of whether the AE was considered related to treatment. Adverse events were classified as non-serious or serious. A serious adverse event (SAE) was any AE that resulted in death, was life-threatening, or resulted in persistent or significant disability/incapacity.

In addition to overall incidence, AEs were also categorized by likelihood of causality as assessed by the veterinarian (probable, possible, unlikely, unclassifiable, or inconclusive) according to VICH GL9 regulations. To better categorize events, AEs were also divided chronologically into those occurring within 24 h of treatment or after 24 h.

Complete blood count, serum biochemistry, and urinalysis were collected at screening, day 0 (if more than 30 days had elapsed since screening), day 14, day 90, and at any unscheduled visits at the discretion of the veterinarian. Samples were analyzed at a central diagnostic laboratory. Values outside reference ranges were classified by the veterinarian as clinically significant or not clinically significant.

### 2.5 Owner and veterinarian outcome assessments

Owner and veterinarian assessments were performed at screening (day −14 to 0), baseline (day 0; treatment 1), day 14 (treatment 2), day 30, day 60, and day 90. Overnight hospitalization following treatment on days 0 and 14 was optional if deemed appropriate by the clinical investigator. Hospitalized cats underwent a physical examination the following day (days 1 and 15), whereas cats discharged the day of treatment were monitored via an owner phone interview the evening of treatment and the day following treatment.

In addition to the CSOM, owners completed an Owner Overall Assessment (OOA) in which they evaluated their cat’s overall response to treatment using a categorical scale (greatly improved, mildly improved, no change, or worsened). Treatment success was defined by a response of “mildly improved” or “greatly improved.” This outcome measure is accepted by the United States Federal Drug Administration Center for Veterinary Medicine (FDA-CVM) and has been previously published as an assessment tool for MSC therapy in cats (15).

In addition to the VPA, veterinarians completed an assessment of overall response to treatment graded on a four-point categorical scale: excellent (clinical signs of OA eliminated or reduced to an inconsequential level), good (clinical signs substantially [at least 50%] reduced), fair (clinical signs minimally [less than 50%] reduced), or poor (clinical signs unaffected by therapy). Treatment success was defined as a score of “good” or “excellent,” whereas “fair” or “poor” scores were considered treatment failures.

### 2.6 Statistical analysis

The study was designed to enroll a sufficient number of cats to achieve a per-protocol evaluable population of 10 cats in each UMSC treatment group (20 UMSC-treated cats total) and 10 placebo-treated cats. This sample size was considered adequate to detect an efficacy signal across the measured outcomes, evaluate potential differences between the UMSC dose groups, and assess safety. A formal power analysis was not performed, and the study was not designed to detect statistically significant differences. Descriptive statistics were used to describe the study population. Continuous clinical pathology variables were summarized using means, standard errors, ranges, and 90% confidence intervals and were interpreted relative to individual baseline values (defined as day 0 values, or screening values if not assessed on day 0) and applicable central diagnostic laboratory reference ranges.

Cats that received at least one dose of study treatment were included in the safety population. The evaluable efficacy population was determined on a case-by-case basis prior to unmasking based on relevant protocol deviations for each outcome parameter. For each outcome measure, the proportion of evaluable cats achieving predefined treatment success at each timepoint was summarized, with a two-sided 90% confidence interval calculated using the exact (Clopper–Pearson) binomial method.

## 3 Results

**Figure 2:**
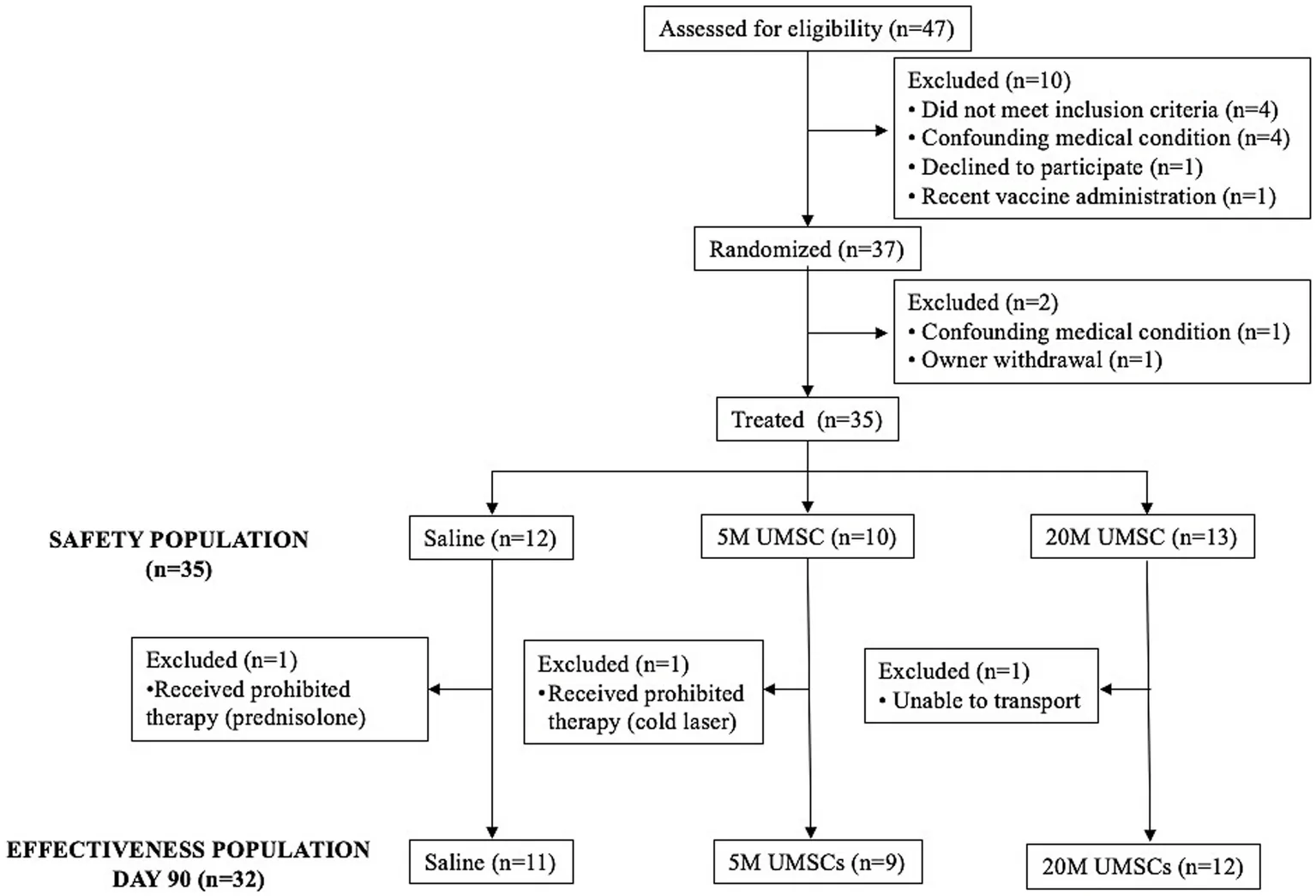
Evaluable population of cats with osteoarthritis receiving 5 million mesenchymal stromal cells (MSCs), 20 million MSCs, or saline placebo intravenously.

Forty-seven cats were screened for eligibility, of which 37 were enrolled and randomized (Figure 2). Two cats were withdrawn after randomization but prior to treatment, leaving a total of 35 cats in the treated safety population: 13 in the 20 M UMSC group, 10 in the 5 M UMSC group, and 12 in the saline placebo group.

### 3.1 Study population

Signalment data for the three groups is reported in Table 1. Of the 35 cats in the safety population, the mean age was 12.7 years (range, 4–19 years) and mean body weight was 5.6 kg (range, 2.9–8.7 kg). The total population included 16 domestic shorthairs, seven domestic medium hairs, three each of domestic longhairs, Maine Coons and American shorthairs, and one each of Persian, Himalayan, and Snowshoe. There were 24 female spayed cats and 11 male neutered cats.

**Table 1:** Signalment characteristics of cats with osteoarthritis receiving 5 million uterine-derived mesenchymal stromal cells (5 M UMSCs), 20 million MSCs (20 M UMSCs), or saline placebo intravenously.

Treatment group	Age (years) Mean +/− SD (Range)	Weight (Kg) Mean +/− SD (Range)	BCS Mean +/− SD (Range)	Sex	Breed (number)
Saline control	12.8 +/−2.89 (9–19)	5.84 +/− 1.38 (3.8–8.7)	6.1 +/− 1.15 (4–8)	10 FS, 2 MN	AS (1), DLH (2), DMH (1), DSH (4), H (1), MC (2), S (1)
5 M UMSCs	11.5 +/−3.31 (4–16)	5.42 +/− 1.29 (3.6–7.6)	5.6 +/− 1.07 (4–7)	4 FS, 6 MN	AS (1), DMH (3), DSH (4), MC (1), P (1)
20 M UMSCs	13.6 +/− 3.23 (9–19)	5.54 +/− 1.67 (2.9–8.5)	5.3 +/−1.38 (3–7)	10 FS, 3 MN	AS (1), DLH (1), DMH (3), DSH (8)

AS, American Shorthair; BCS, Body condition score; DLH, Domestic Longhair; DMH, Domestic Mediumhair; DSH, Domestic Shorthair; FS, Female Spayed; H, Himalayan; M, Million; MC, Maine Coon; MN, Male Neutered; P, Persian; S, Snowshoe; SD, Standard deviation; UMSCS, Uterine-derived mesenchymal stromal cells.

The mean CSOM at Day 0 was similar between groups: 7.1 (range 3–11) in the saline group, 7.8 (range 5–12) in the 5 M UMSC group, and 7.8 (range 4–12) in the 20 M UMSC group. One cat in the saline group and one cat in the 20 M UMSC group had an improved CSOM between screening and day 0, accounting for the minimum of 3 and 4 in those groups despite all cats meeting inclusion criteria for a minimum CSOM of 5 at screening. Categorization of the CSOM by activity type prior to treatment is represented in Table 2. The most common abnormal activities included difficulty jumping and climbing up and reduced play activity. Change in gait or lameness was reported much less commonly.

**Table 2:** Client specific outcome measure (CSOM) activities prior to treatment in cats with naturally occurring osteoarthritis (categorized).

Clinical sign	Percentage of selected activities
Difficulty jumping up	37%
Reduced play activity	18%
Difficulty climbing up	16%
Difficulty jumping down	8.5%
Difficulty rising/stiffness	5.4%
Change in gait/lameness	4.6%
Difficulty getting in/out of the litterbox, change in litterbox habits	3.8%
Reduced movement/flexibility	2.3%
Change in socialization/behavior	1.5%
Reduced grooming	0.7%
Change in sleeping location	0.7%
No longer sitting by feeder when ready for food	0.7%

Veterinarians scored pain in up to four joints for each cat, and the mean pain scores at screening for each joint are listed in Table 3. Seventy one percent of cats had at least three joints involved and 43% had four joints involved. The treatment groups were well balanced at baseline, with similar numbers of affected joints as well as comparable mean, minimum, and maximum pain scores, indicating that the study populations were similar before treatment.

**Table 3:** Veterinarian pain score in evaluated joints in each treatment group prior to treatment (maximum score of 5).

Treatment Group	Affected Joint Number	Number of cats	Mean pain score +/− SD	Minimum pain score	Maximum pain score
Saline	Joint 1	12	3.17 +/− 0.83	2	4
	Joint 2	12	3.25 +/− 0.75	2	4
	Joint 3	8	2.38 +/− 0.92	1	4
	Joint 4	4	2.5 +/−1.29	1	4
5 M UMSCs	Joint 1	10	3.2 +/− 0.63	2	4
	Joint 2	10	3.3 +/− 0.48	3	4
	Joint 3	8	2.25 +/− 0.89	1	3
	Joint 4	5	2.4 +/− 0.89	1	3
20 M UMSCs	Joint 1	13	3.31 +/− 0.63	2	4
	Joint 2	13	3 +/− 0.91	2	5
	Joint 3	9	2.89 +/−1.17	1	4
	Joint 4	6	3 +/− 1.1	1	4

5 M UMSCs, 5 million uterine derived mesenchymal stromal cells; 20 M UMSCs, 20 million uterine derived mesenchymal stromal cells; SD, Standard Deviation.

The percentage of cats with specific joint involvement at screening is listed in Table 4. The stifles, hips, and elbows were the most commonly affected joints across all treatment groups. Hip involvement was more common in cats receiving 5 M UMSCs, whereas stifle involvement was more common in the 20 M UMSC and placebo groups.

**Table 4:** Affected joints within each treatment group prior to treatment.

Joint	5 M UMSCs (*n* = 10) % of cats	20 M UMSCs (*n* = 13) % of cats	Saline placebo (*n* = 12) % of cats %)	Total group (*n* = 35) % of cats
Stifle	40%	84.6%	75%	68.6%
Hip	90%	61.5%	50%	65.7%
Elbow	40%	61.5%	75%	60%
Shoulder	30%	7.7%	8.3%	14.3%
Lumbosacral	20%	0%	25%	14.3%

5 M UMSCs, 5 million uterine derived mesenchymal stromal cells; 20 M UMSCs, 20 million uterine derived mesenchymal stromal cells.

### 3.2 Safety assessment

Thirty-four cats received two doses of UMSCs or saline per protocol; one 20 M UMSC dose cat received one dose because the owner was unable to get the cat in the carrier for the second dose and withdrew from the study. Concomitant medications administered prior to catheter placement included: gabapentin (four saline, three 5 M UMSC, five 20 M UMSC cats), maropitant (three saline, four 20 M UMSC cats), pregabalin (two 20 M UMSC cats), and butorphanol (two 5 M UMSC cats).

The number of cats with AEs reported throughout the 90-day study are summarized in Table 5. The percentage of non-serious AEs possibly or probably associated with treatment was lowest in the 5 M UMSC group (10%). There were no SAEs possibly or probably associated with treatment in the 5 M UMSC or saline groups and one SAE possibly or probably associated with treatment in the 20 M UMSC group.

**Table 5:** Adverse events (AEs) in cats reported through day 90.

Treatment group (*n* = number of cats)	Adverse event (AE) type	Total number of cats with AE (%)	Number of cats (%) with AE possibly/probably associated with treatment
Saline (*n* = 12)	Non-serious	67%	17%
	Serious	8.3%	0%
	Total	75%	17%
5 M UMSCs (*n* = 10)	Non-serious	60%	10%
	Serious	0%	0%
	Total	60%	10%
20 M UMSCs (*n* = 13)	Non-serious	54%	31%
	Serious	15%	7.8%
	Total	69%	38%

Adverse events where causality was possibly or probably associated with treatment that were reported within 24 h of treatment are summarized in Table 6 and represent five cats in the 20 M UMSC dose group and one cat each in the 5 M UMSC dose and saline groups. Only one cat exhibited AEs with both treatments (Day 0 and Day 14). Adverse events were most commonly associated with transient nausea or gastrointestinal signs or transient weakness and lethargy.

**Table 6:** Adverse events reported within 24 h of treatment with uterine derived mesenchymal stromal cells (UMSCs) or placebo in cats with naturally occurring osteoarthritis.

Adverse reaction	Number of cats^a^ (20 M group)	Number of cats^a^ (5 M group)	Number of cats (Saline group)
Hypersalivation	2	0	0
Muscle weakness	2	0	0
Lethargy	2	0	0
Malodor	2	0	0
Nausea/Vomiting	2	0	0
Nystagmus	1	1	0
Tachypnea	1	1	0
Diarrhea	0	0	1
Inappropriate defecation	1	0	0
Injection site inflammation	1	0	0

a More than one clinical sign could be attributed to a single cat.

Three SAEs occurred during the 90 days study, one in the saline group and two in the 20 M UMSC group; only one of these cases was considered treatment related, and this was the only cat to receive overnight hospitalization. In the one AE thought to be treatment related, the cat demonstrated mild lethargy, nausea, and hypersalivation after administration of the first dose, was treated with maropitant, and recovered uneventfully. At the second dose administration 14 days later, the same cat showed weakness, tachypnea, vocalization, and nystagmus during the injection. The cat was treated with dexamethasone, maropitant, ondansetron, and oxygen, the recovered uneventfully and completed the 90-day study. No SAEs were reported in the 5 M UMSC group.

### 3.3 Clinical pathology data

Complete CBC, biochemistry panel including electrolytes, liver, renal and muscle enzymes, and urinalysis were obtained at screening and days 14 and 90 for all 35 cats. In the saline group, all bloodwork values remained stable or improved throughout the study with the exception of progressive hypercalcemia and hypermagnesemia at day 14 in one cat and mild neutrophilia in one cat at day 90. In the 5 M dose group, all bloodwork values remained stable or improved throughout the study with the exception of a mild elevation in creatinine in one cat at day 14 but not at day 90 and mild elevations in globulin and ALT in one cat each at day 90. In the 20 M group, all bloodwork values remained stable or improved throughout the study with the exception of mild hyperglycemia in two cats and mild anemia in one cat at day 90.

### 3.4 Owner and veterinarian outcome assessments

Of the 35 cats enrolled and treated, 32 cats were evaluable for at least one efficacy outcome at days 14, 30, 60, and 90. Owner assessment was not available for one cat at any timepoint because the owner did not fill out the assessment forms. Two cats were not evaluable at Day 14 because owners filled out the assessment forms too late. Two cats were not evaluable at Day 30 because one saline-treated cat was removed due to administration of a non-study related prohibited therapy (prednisolone for pre-existing dermatitis), and the assessment was filled out by a different owner in one cat. Two cats were not evaluable at day 60 because one saline-treated cat exited the study because the owner was concerned about the cat’s level of discomfort, and one 5 M UMSC dose cat was removed due to administration of a prohibited therapy (cold laser treatment). One cat in the 5 M UMSC dose group was missing a result for the veterinarian assessments on day 90 because the exam was not completed by the same veterinarian. Thirty-one cats were evaluable for all four efficacy outcomes at day 90.

Efficacy data for evaluable cats for each outcome (CSOM, OOA, VPA, and VOA) in the 5UMSC, 20 M UMSC, and saline groups at days 14, 30, 60, and 90 with 90% confidence intervals is listed in Table 7. The percentage of cats classified as treatment successes increased progressively over the course of the study in both UMSC-treated groups but remained relatively unchanged in the placebo group. By Day 90, at least 75% of cats receiving 5 M or 20 M UMSCs met the treatment success criteria for CSOM, OOA, and VPA, compared with 36% of placebo-treated cats for CSOM and OOA and 45% for VPA. Success rates based on VOA were at least 50% in the treated groups compared to 20% in the placebo group.

**Table 7:** Client specific outcome measures (CSOM), owner overall assessment (OOA), veterinarian pain assessment (VPA), veterinarian overall assessment (VOA) and success at days 14, 30, 60, 90 in cats treated with intravenous uterine derived mesenchymal stromal cells (UMSCs) at 5 million (5 M) or 20 million (20 M) cells versus saline control.

Outcome	Group	Day 14	90% CI	Day 30	90% CI	Day 60	90% CI	Day 90	90% CI
CSOM	5 M UMSCs	5/10 (50%)	22–78%	5/10 (50%)	22–78%	8/10 (80%)	49–96%	7/9 (78%)	45–96%
	20 M UMSCs	6/10 (60%)	30–85%	7/11 (64%)	35–86%	10/12 (83%)	56–97%	10/12 (83%)	56–97%
	Control	4/12 (33%)	12–61%	5/11 (45%)	20–73%	6/10 (60%)	30–85%	4/11 (36%)	14–65%
OOA	5 M UMSCs	8/10 (80%)	49–96%	9/10 (90%)	61–99%	7/10 (70%)	39–91%	7/9 (78%)	45–96%
	20 M UMSCs	7/10 (70%)	39–91%	9/11 (82%)	53–97%	8/12 (67%)	39–88%	10/12 (83%)	56–97%
	Control	4/12 (33%)	12–61%	6/11 (55%)	27–80%	6/10 (60%)	30–85%	4/11 (36%)	14–65%
VPA	5 M UMSCs	8/10 (80%)	49–96%	7/9 (78%)	45–96%	7/9 (78%)	45–96%	6/8 (75%)	40–95%
	20 M UMSCs	6/12 (50%)	25–75%	8/11 (73%)	44–92%	10/12 (83%)	56–97%	10/12 (83%)	56–97%
	Control	6/10 (60%)	30–85%	6/11 (55%)	27–80%	4/9 (44%)	17–75%	5/11 (45%)	20–73%
VOA	5 M UMSCs	2/10 (20%)	4–51%	3/8 (38%)	11–71%	5/9 (56%)	25–83%	4/8 (50%)	19–81%
	20 M UMSCs	1/12 (8%)	0–34%	5/10 (50%)	22–78%	6/12 (50%)	25–75%	8/12 (67%)	39–88%
	Control	1/10 (10%)	1–39%	1/12 (8.3%)	0–34%	2/10 (20%)	4–51%	2/10 (20%)	4–51%

5 M, 5 million; 20 M, 20 million; CI, Confidence interval; CSOM, Client Specific Outcome Measures; OOA, Owner overall assessment; UMSCs, Uterine-derived mesenchymal stromal cells; VPA, Veterinarian pain assessment; VOA, Veterinarian overall assessment.

**Figure 3:**
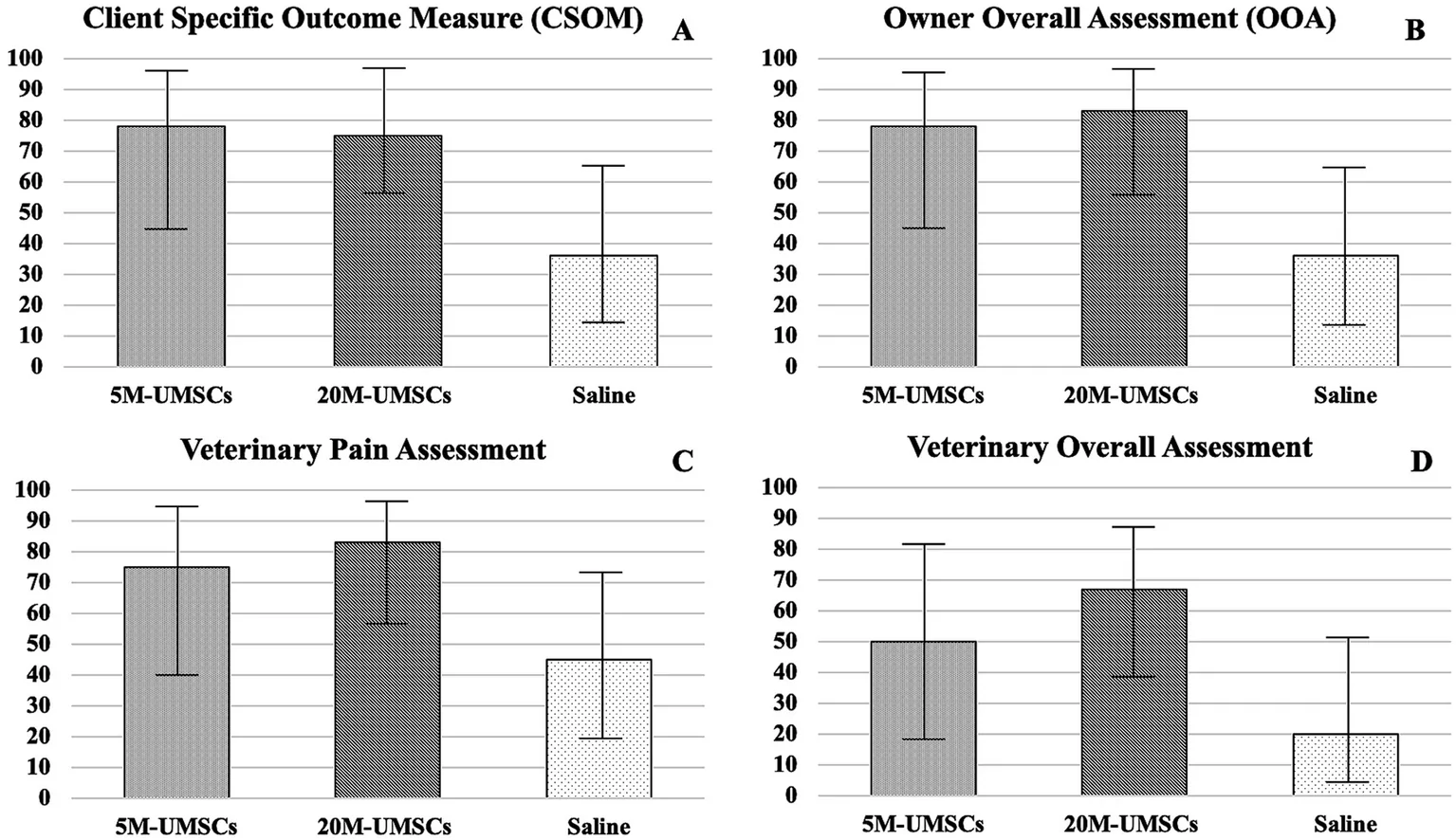
Percentage of cats considered a treatment success at day 90 after receiving 5 million (5 M) or 20 million (20 M) uterine-derived mesenchymal stromal cells (UMSCs) versus saline placebo. Client Specific Outcome Measures **(A)**, Owner Overall Assessment **(B)**, Veterinarian Pain Assessment **(C)**, Veterinarian Overall Assessment **(D)**. 5 M-UMSCS, 5 million uterine-derived mesenchymal stromal cells; 20 M-UMSCS, 20 million uterine-derived mesenchymal stromal cells.

Figure 3 outlines the number of cats deemed treatment successes for each outcome. Treatment success was higher for the 5 M and 20 M UMSC groups compared to placebo for all outcome measures at day 90 with success in >75% of cats in the UMSC treated groups for CSOM, OOA, and VPA.

**Figure 4:**
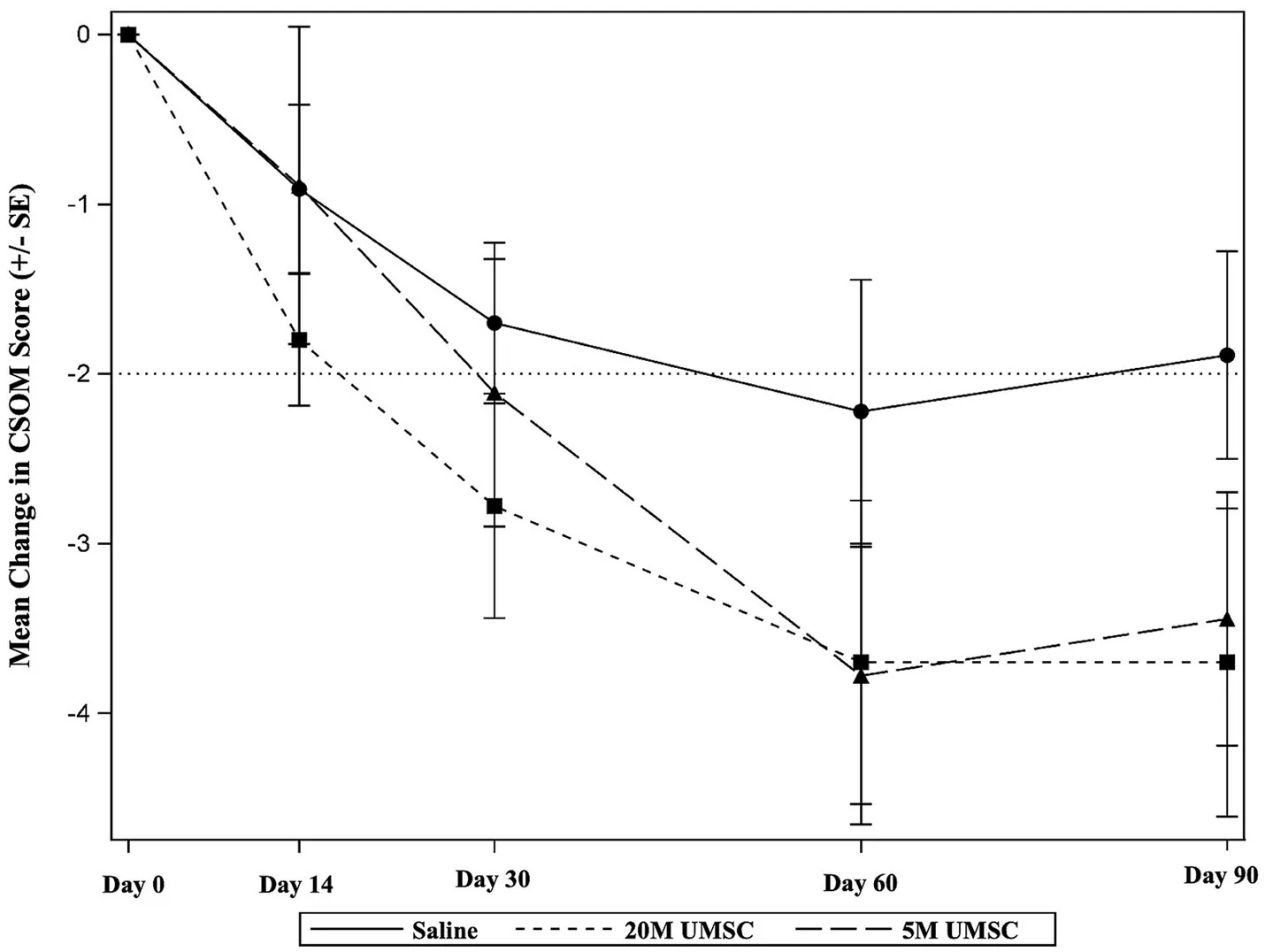
Mean change in the client specific outcome measures (CSOM) score at day 14, 30, 60, and 90 in cats with osteoarthritis treated with 5 million (5 M) or 20 million (20 M) uterine-derived mesenchymal stromal cells (UMSCs) compared to saline.

The mean change in CSOM was assessed for the 5 M UMSC, 20 M UMSC and saline treated groups (Table 8; Figure 4). The mean change was largest for the 5 M and 20 M UMSC groups, >3.5 at days 60 and 90 for both treatment groups compared to a mean change of 1.9 in the placebo group (minimum change of 2 considered a treatment success in each patient).

**Table 8:** Mean change in client specific outcome measure in cats receiving 5 million (5 M) uterine-derived MSCs (UMSCs) or 20 million (20 M) UMSCs compared to saline placebo.

Day	Saline mean improvement (+/− SE)	5 M UMSCs mean improvement (+/− SE)	20 M UMSCs mean improvement (+/− SE)
14	0.9 +/− 0.49	0.9 +/− 0.39	1.8 +/− 0.93
30	1.7 +/− 0.47	2.1 +/− 0.66	2.8 +/− 0.79
60	2.2 +/−0.78	3.8 +/− 0.96	3.7 +/− 0.76
90	1.9 +/− 0.61	3.5 +/− 0.91	3.7 +/− 0.75

SE, standard error; 5 M UMSCs, 5 million uterine derived mesenchymal stromal cells; 20 M UMSCs, 20 million uterine derived mesenchymal stromal cells.

## 4 Discussion

In this pilot study, UMSCs were well tolerated and showed improved outcomes in validated owner assessed parameters as well as veterinarian outcomes in greater than or equal to 75% of cats at both 5 M and 20 M UMSC dosing. These findings suggest that IV MSC therapy may have a minimum 3-month efficacy (study end) for control of clinical signs associated with naturally occurring OA in cats.

Feline OA is a chronic, low-grade inflammatory disease that often manifests with subtle behavioral and lifestyle changes (4). The cats in this study appeared representative of the typical feline OA population, including predominantly middle-aged to older cats with multifocal joint involvement and CSOM changes consistent with alterations in jumping, climbing, and play activity, with very few showing observed lameness (3, 33, 38, 39). The involved joints were similar to previous reports, with stifle, hip and elbow being most common (38).

Intravenous allogeneic MSC administration is particularly appealing for regenerative medicine therapy in the feline OA population given that it negates the need for anesthesia and invasive autologous cell harvest, avoids the technical challenges of intra-articular (IA) MSC administration into small joints, and minimizes the potential risks of iatrogenic infection and transient exacerbation of joint inflammation (40). The single IV injection in this study was easily administered, increasing the feasibility and accessibility of MSC therapy. In addition to targeting the systemic drivers of immune dysregulation, the natural homing mechanisms of MSCs allow them to migrate directly to sites of inflammation when administered IV (16, 27, 41). Intravenous administration is particularly appealing in cats, where multi-joint OA is common (2, 3, 38, 42).

UMSC therapy was well tolerated in this study population. These safety findings broadly align with previous IV MSC trials in cats (15, 16, 24, 43). Repeat dosing of intravenous allogeneic MSCs has been employed in many feline studies (13, 15–17, 44), with no reported safety concerns related to immunogenicity in long term follow-up studies (14, 44, 45). In the 5 M dose group, treatment-related adverse events occurred in only 10% of cats (1 of 20 (5%) of doses), and there were no SAEs. The most likely time to observe a treatment related AE with UMSCs was during the injection or within 24 h following treatment, and these AEs were mild and resolved uneventfully. The most common AEs were related to nausea and lethargy, consistent with previously reported AEs with IV allogeneic MSC administration in cats (13, 15–17, 44). The cause of these AEs and relation to the MSCs themselves is still largely unknown (24, 46).

There was one SAE associated with treatment in the 20 M dose group and represents a possible hypersensitivity reaction, potentially related to the formulation and not the cells themselves given that adverse events including vomiting and increased respiratory rate and effort are reported in association with DMSO-preserved cells (43). Other products currently available for the management of feline OA include NSAIDs or anti-NGF monoclonal antibodies, which may be associated with off-target toxicity in some cats (10, 47, 48). As systemic inhibitors of prostaglandins, NSAIDs may cause AEs involving the gastrointestinal tract, kidneys, and liver, contributing to the lack of currently FDA-approved NSAIDs for long-term use in cats (49). MSC therapy may offer a targeted and safe option for cats with OA.

Evaluating the efficacy of an OA therapy for cats requires an outcome measure focused on daily mobility and quality of life. CSOM is ideal for this purpose, as it allows owners to quantify changes in their cat’s unique daily routines on an individual and subjective bases (33). CSOM is a validated parameter for assessing OA in cats and has been recently used as a primary efficacy endpoint for the regulatory approval of an anti-NGF monoclonal antibody for pain in cats with OA (11, 50). In the present study, this validity is reinforced by the owner overall assessment success rates, which closely paralleled the CSOM success rates. The mean change in CSOM score was higher in the UMSC treated groups compared to placebo for all timepoints, with a distinction at day 60 day that was maintained through day 90. A longer duration of efficacy is possible and has been demonstrated for MSCs in other indications (14, 45, 51). The placebo effect appeared to be less pronounced in this study than has been reported in previous feline OA trials, where it is a well-recognized limitation (52). In a frunevetmab study, CSOM treatment success at a comparable post-treatment time point (Day 84) was reported in 76% of treated cats, but also in 68% of placebo-treated cats (35).

The clinical improvements observed in both owner and veterinarian assessments in this study suggest that the immunomodulatory mechanisms of MSCs may mitigate the systemic and local inflammatory processes and immune dysregulation in cats with OA, translating to improved patient mobility and quality of life (5, 25). Reduced pain and inflammation following UMSC therapy may result from decreased production of proinflammatory mediators such as tumor necrosis factor-alpha (TNF-α) and interleukin-2 (IL-2), likely mediated through immunomodulation and cell-to-cell signaling by systemically administered UMSCs (5, 53). The immunomodulatory and potentially disease modifying effects differentiate MSCs from other currently available therapeutic options, which reduce clinical signs by temporarily dampening pain signals or inflammation but do not target the root cause of disease (54, 55).

This study carries the inherent limitations of a pilot study with approximately 10 cats in each group, contributing to a wide confidence interval that should be acknowledged when noting differences between the UMSC-treated and control groups. Owner-completed assessment is inherently susceptible to the caregiver placebo effect, a well-documented phenomenon in feline OA trials; however, there was a clear difference in outcomes between placebo and UMSC treated cats in this study for all outcomes (4, 11). The 90-day study duration limits assessment of efficacy at later timepoints and warrants further evaluation based on the positive outcomes at 3 months.

This pilot study demonstrated that IV allogeneic UMSC administration was well tolerated and showed efficacy for cats with OA at both evaluated doses. The randomized, masked, placebo-controlled design and adherence to clinically relevant inclusion criteria in a naturally occurring OA population support generalizability to real-world clinical practice. The low number of AEs and relatively equivalent efficacy of the 5 M UMSC dose compared to the 20 M UMSC dose supports the design of a larger long-term placebo-controlled clinical field trial evaluating 5 M UMSCs in cats with naturally occurring OA.

## Data Availability

The datasets presented in this article are not readily available because they contain proprietary information and are part of an FDA regulated study. Requests to access the datasets should be directed to rebeccaw@gallant.com.
